# Alarming Status of the Decayed, Missing, and Filled teeth (DMFT) Index in Iran: Challenges and Solutions

**DOI:** 10.34172/aim.34930

**Published:** 2026-02-01

**Authors:** Sanaz Amiri, Seyed Parsa Dehghani, Habibollah Azarbakhsh

**Affiliations:** ^1^Student Research Committee, Shiraz University of Medical Sciences, Shiraz, Iran; ^2^School of Medicine, Shiraz University of Medical Sciences, Shiraz, Iran; ^3^Department of Community Medicine, School of Medicine, Ahvaz Jundishapur University of Medical Sciences, Ahvaz, Iran

## Dear Editor

 Oral health is a vital component of overall health and has a direct impact on individual well-being.^1^ Poor oral health adversely affects the quality of life and general well-being.^2^ The DMFT index is one of the practical indices for assessing oral health, which includes the number of Decayed, Missing, and Filled Teeth in an individual.^3^ While global prevalence rates of DMFT are estimated at 46.2%, recent national data from Iran indicate a DMFT prevalence of 53.8%.^4^ This comparison highlights that Iran bears a greater burden than the global average, emphasizing the necessity for targeted national strategies and increased awareness.

 The indicator affects individuals across all age groups, with studies reporting concerning levels in both younger and older populations.

 Iran continues to face a troubling oral health burden, with DMFT scores among children and adults remaining above global targets. Recent national data show that the average DMFT score for 12-year-olds exceeds 3.0, and adult populations report even higher levels—reflecting persistent gaps in prevention, access, and policy implementation.^5^ Approximately 22% of Iranian children’s primary teeth were affected by dental caries in 1990, of which over 83% were decayed [3.64] and fewer than 7% were filled. Between 1990 and 2017, the DMFT index increased by more than 15%.^6^ Among Iranians aged 15 to 40 years, the average numbers of decayed teeth (DT), missing teeth (MT), and filled teeth (FT) were estimated at 2.58 ± 1.70, 1.84 ± 1.15, and 1.7 ± 3.35, respectively. The overall average DMFT index was estimated at 7.33.^7^ For individuals aged 40 to 70 years, the mean and standard deviation of the DMFT index were reported as 18.06 ± 8.70.^8^

 Moreover, between 1990 and 2017 based on literature search, the average DMFT index across all age groups increased by approximately 58% (from 6.8 in 1990 to 10.8 in 2017). During this period, decayed teeth (DT) and missing teeth (MT) increased by 84.5% and 31.6%, respectively. Filled teeth (FT) increased by nearly 2.6 times, from 0.6 in 1990 to 1.7 (95% UI, 0.6–2.8) in 2017. The DT-to-FT ratio continuously increased in both genders. In 2017, the highest DT, MT, and FT values were observed in the 25–29 (4.9), 60 + (21.5), and 35–39 (2.6) age groups, respectively.^9^ Hence, permanent tooth decay imposes an increasing burden on the Iranian population. The high prevalence and experience of dental caries in Iran appears to indicate ineffective national preventive programs in the field of oral health and lack of educational measures.

 Despite the availability of national oral health guidelines, Iran lacks a coordinated implementation strategy, particularly in underserved regions. The absence of school-based preventive programs and limited access to fluoride interventions contribute to the persistently high DMFT scores.^10^ To reduce this burden, several solutions can be proposed, broadly categorized into individual/behavioral and health system interventions.

## Individual and Behavioral Interventions, Raising Public Awareness

 Launching educational campaigns about oral hygiene in schools, media, and health centers.^11^ Maintaining oral hygiene: Brushing at least twice daily with fluoride toothpaste and using dental floss daily.^12^ Using fluoride mouthwashes: To strengthen enamel and reduce bacterial activity. Regular dental check-ups: Periodic visits for early detection and preventive care.^13^

## Health System Interventions

###  Community fluoride therapy

 Adding fluoride to drinking water in deficient areas or providing fluoride mouthwashes in schools. Studies have shown that the amount of fluoride in water in most regions of Iran is less than the required amount.^14^

###  Free or subsidized preventive services

 Such as dental sealants for children and adolescents at health centers.

###  Training for dentists and healthcare providers

 Emphasizing prevention over mere treatment.

###  Monitoring and recording epidemiological data

 To identify high-risk areas and allocate targeted resources.

 Insurance coverage for dental services. As there is a greater focus on the use of dental services among households with higher socioeconomic status in Iran, inequality favors the wealthy in the use of dental services.^15^

## References

[1] Chestnutt IG. Dental Public Health at a Glance. John Wiley & Sons; 2024.

[2] Spanemberg JC, Cardoso JA, Slob E, López-López J (2019). Quality of life related to oral health and its impact in adults. J Stomatol Oral Maxillofac Surg.

[3] Villavicencio-Caparo E, Alvarracin-Duran AJ (2025). Association between body mass index and decayed, missing, and filled teeth (DMFT) in 12-year-old school children. Open Dent J.

[4] Maklennan A, Borg-Bartolo R, Wierichs RJ, Esteves-Oliveira M, Campus G (2024). A systematic review and meta-analysis on early-childhood-caries global data. BMC Oral Health.

[5] Ghasemianpour M, Bakhshandeh S, Shirvani A, Emadi N, Samadzadeh H, Moosavi Fatemi N (2019). Dental caries experience and socio-economic status among Iranian children: a multilevel analysis. BMC Public Health.

[6] Shoaee S, Saeedi Moghaddam S, Masinaei M, Sofi-Mahmudi A, Hessari H, Heydari MH (2022). Trends in dental caries of deciduous teeth in Iran: a systematic analysis of the national and sub-national data from 1990 to 2017. BMC Oral Health.

[7] Moradi G, Mohamadi Bolbanabad A, Moinafshar A, Adabi H, Sharafi M, Zareie B (2019). Evaluation of oral health status based on the decayed, missing and filled teeth (DMFT) index. Iran J Public Health.

[8] Ghoddusi Johari M, Moftakhar L, Rahimikazerooni S, Rezaeianzadeh R, Hosseini SV, Rezaianzadeh A (2021). Evaluation of oral health status based on DMF index in adults aged 40-70 years: findings from Persian Kharameh cohort study in Iran. J Dent (Shiraz).

[9] Shoaee S, Masinaei M, Saeedi Moghaddam S, Sofi-Mahmudi A, Hessari H, Shamsoddin E (2024). National and subnational trend of dental caries of permanent teeth in Iran, 1990-2017. Int Dent J.

[10] Soltani MR, Sayadizadeh M, Raeisi Estabragh S, Ghannadan K, Malek-Mohammadi M (2020). Dental caries status and its related factors in Iran: a meta-analysis. J Dent (Shiraz).

[11] Armoon B, Yazdanian M, Higgs P, Sanaei Nasab H (2021). Effect of a hospital-based oral health-education program on Iranian staff: evaluating a theory-driven intervention. BMC Med Educ.

[12] Cui Z, Wang W, Si Y, Wang X, Feng X, Tai B (2023). Tooth brushing with fluoridated toothpaste and associated factors among Chinese adolescents: a nationwide cross-sectional study. BMC Oral Health.

[13] Khodavirdizadeh Ghahremani S, Ghasemi Shayan R, Kia SJ, Khosousi Sani S, Khodavirdizadeh Ghahremani G. Oral health, DMFT and disease prevalence among HIV-positive patients in Tabriz, Iran in 2024. Res Sq [Preprint]. December 18, 2024. Available from: https://www.researchsquare.com/article/rs-5453446/v1.

[14] Yari AR, Nazari S, Mahvi AH, Majidi G, Alizadeh Matboo S, Fazlzadeh M (2016). Fluoride concentration of drinking-water of Qom, Iran. Iran J Health Sci.

[15] Rezaei S, Hajizadeh M, Irandoost SF, Salimi Y (2019). Socioeconomic inequality in dental care utilization in Iran: a decomposition approach. Int J Equity Health.

